# Repeated Awake Surgical Resection(s) for Recurrent Diffuse Low-Grade Gliomas: Why, When, and How to Reoperate?

**DOI:** 10.3389/fonc.2022.947933

**Published:** 2022-07-05

**Authors:** Hugues Duffau

**Affiliations:** ^1^Department of Neurosurgery, Gui de Chauliac Hospital, Montpellier University Medical Center, Montpellier, France; ^2^Team “Plasticity of Central Nervous System, Stem Cells and Glial Tumors”, National Institute for Health and Medical Research (INSERM), U1191 Laboratory, Institute of Functional Genomics, University of Montpellier, Montpellier, France

**Keywords:** brain connectome, electrostimulation mapping, low-grade glioma, multistage management, neuroplasticity, quality of life, reoperation, awake brain surgery

## Abstract

Early maximal surgical resection is the first treatment in diffuse low-grade glioma (DLGG), because the reduction of tumor volume delays malignant transformation and extends survival. Awake surgery with intraoperative mapping and behavioral monitoring enables to preserve quality of life (QoL). However, because of the infiltrative nature of DLGG, relapse is unavoidable, even after (supra)total resection. Therefore, besides chemotherapy and radiotherapy, the question of reoperation(s) is increasingly raised, especially because patients with DLGG usually enjoy a normal life with long-lasting projects. Here, the purpose is to review the literature in the emerging field of iterative surgeries in DLGG. First, long-term follow-up results showed that patients with DLGG who underwent multiple surgeries had an increased survival (above 17 years) with preservation of QoL. Second, the criteria guiding the decision to reoperate and defining the optimal timing are discussed, mainly based on the dynamic intercommunication between the glioma relapse (including its kinetics and pattern of regrowth) and the reactional cerebral reorganization—i.e., mechanisms underpinning reconfiguration within and across neural networks to enable functional compensation. Third, how to adapt medico-surgical strategy to this individual spatiotemporal brain tumor interplay is detailed, by considering the perpetual changes in connectome. These data support early reoperation in recurrent DLGG, before the onset of symptoms and before malignant transformation. Repeat awake resection(s) should be integrated in a global management including (neo)adjuvant medical treatments, to enhance long-lasting functional and oncological outcomes. The prediction of potential and limitation of neuroplasticity at each step of the disease must be improved to anticipate personalized multistage therapeutic attitudes.

## Introduction

Early and maximal surgical resection currently represents the first treatment in diffuse low-grade glioma (DLGG), because the reduction of tumor volume enables to delay malignant transformation (MT) and to significantly extend the overall survival (OS) (1–6). This is particularly true following supratotal resection (i.e., with the removal of a security margin beyond the Fluid Attenuated Inversion Recovery FLAIR hypersignal visible on preoperative Magnetic Resonance Imaging MRI), because most patients are still alive after long-term follow-up (FU) (7–9). Beyond oncological considerations, awake surgery with intraoperative electrical mapping of neural networks critical for brain functions combined with real-time monitoring of conation, language, cognition, and emotion resulted in the preservation of quality of life (QoL) (10–12) or even in its improvement, especially in case of preoperative epilepsy with seizure freedom following glioma removal (13, 14). Indeed, recent series with accurate postsurgical neurological and neuropsychological examination reported a severe permanent deficit at a rate of about zero and a preservation of neurocognitive functions in most patients (15–17), including after resection of incidental DLGG (18). Furthermore, over 94% of patients were able to resume professional activities, reflecting an actual return to real life following awake surgery in the vast majority of cases (15, 18–20). A perfect illustration of long-lasting project is the desire for motherhood in women with DLGG, mixing the complexity to make such a decision with a chronic brain tumoral disease and the risk of negative impact of pregnancy on glioma behavior (21): A complete resection before being pregnant resulted in a significant longer OS after delivery while maintaining QoL (22).

Despite such drastic improvements of long-term outcomes in DLGG due to an active surgical attitude, because of the invasive feature of this neoplasm, a recurrence is almost unavoidable, even after (supra)total resection (7). Thus, besides chemotherapy and radiotherapy, the question of possible reoperation(s) is increasingly raised, especially because patients with DLGG are usually young and would like to continue to make plans in the long run (23). Here, the purpose is to review the literature in the emerging field of iterative surgeries in recurrent DLGG, regarding the following issues: (i) Did multiple surgeries in DLGG improve life expectancy while sparing QoL? (ii) What are the factors supporting the decision to reoperate and helping to define the optimal timing for subsequent resection? (iii) How to adapt surgical techniques to the individual spatiotemporal brain tumor interplay, based on dynamic interactions between glioma regrowth and reactional neural networks reshaping?

## Why to Reoperate: The Benefit of Repeat Resection(s) on Long-Lasting Outcomes in Patients With DLGG

Despite the paucity of original studies that investigated multiple surgeries in DLGG identified in this qualitative review, namely, 15 series with 630 patients reported between 2003 and 2022, these results support a positive impact of reoperation(s) on long-term oncological and functional outcomes (**Table 1**).

**Table 1:** Original series that reported outcomes following reoperation in patients with DLGG.

First Author (Year)	Number of Patients	Oncological Outcomes	Functional Outcomes
Schmidt et al. (2003) (24)	40	Median time to S2: 22.5–49 months (mean: 3 years) GTR at S1 linked to median time to S2 50% of MT	Not detailed
Martino et al. (2009) (25)	19	Median time to S2: 4.1 years 57% of MT No death with median FU of 6.6 years	Three improvements/three slight deficits Epilepsy control: 84.2% of RTW
Ahmadi et al. (2009) (26)	96	Reoperation correlated to longer OS 44.7% of MT	Not detailed
Kaspera et al. (2013) (27)	16	EOR similar R1 and R2 : 62% of MT	Similar morbidity S1–S2
Ramakrishna et al. (2015) (28)	52	46% of MT OS = 12.95 years EOR at S1 and S2 correlated to OS	8% of permanent deficits
Ius et al. (2015) (29)	23	Median time to S2: 81 months (6.75 years) 74% of MT (insular gliomas) Tumor relapse related to EOR at S1	4.35% of permanent deficits
Southwell et al. (2016) (30)	17	Mean time to S2: 4.1 years	No permanent deficits
Spitael et al. (2017) (31)	25	24% of MT 62% of patients still alive with a median FU of 109 months	Not detailed
Picart et al. (2019) (32)	42	Mean time to S2: 4.1–5 years	No permanent deficits Time to recovery significantly shorter at S2
Zattra et al. (2019) (33)	51	Not detailed	Similar morbidity S1-S2
Morshed et al. (2019) (34)	23	44.9% of MT (insular gliomas)	8.5% of permanent deficits
Shofty et al. (2020) (35)	93	Median time to S2: 38 months (3.2 years) 19% of MT OS = 14.8 years	5% of permanent deficits
Capo et al. (2020) (36)	40	Median time to S2: 49.2 months (4.1 years) 57% of MT 92% of patients still alive at 5 years	No permanent deficits Preservation of cognitive functions
Hamdan et al. (2021) (37)	31	Median time to S2: 4 years Median time S2 to S3: 5.4 years OS = 17.8 years	3.2% of permanent deficits 84.6% of RTW
Ng et al. (2022) (38)	62	Median time to S2: 5.5 years 93.5% of patients still alive after an overall FU of 8.3 years	No permanent deficits Cognition preserved in 93.5% of patients 94.2% of RTW

DLGG, diffuse low-grade glioma; EOR, extent of resection; GTR, gross total resection; MT, malignant transformation; OS, overall survival; RTW, return to work; S1, first surgery; S2, second surgery; S3, third surgery.

### Oncological Considerations

From an oncological perspective, the median survival was not yet reached at the last FU in several cohorts: no death with a median FU from initial diagnosis at 6.6 years in the study of Martino et al. (25); 62% of patients still alive following a median FU of 109 months in the study of Spitaels et al. (31); 92% of patients still alive at 5 years in the study of Capo et al. (36); and 93.5% still alive after an overall FU of 8.3 years in the study of Ng et al. (38). In studies with enough FU to report survival, the OS was 12.95 years in the study of Ramakrishna et al. (28) and 14.8 years in the study of Shofty et al. (35), namely, significantly longer in comparison with an OS of only 6.5 years in a control group of patients who experienced DLGG progression or transformation but who did not undergo a second surgery (p = 0.0001) (35). Similarly, Ahmadi et al. (26) reported a significantly prolonged OS in patients who had a second total resection in case of relapse without MT compared with patients who had a complete resection only once. Remarkably, in a recent consecutive series that detailed long-lasting outcomes after three iterative surgeries, an unprecedented median OS of 17.8 years has been reached (26).

Interestingly, OS is correlated to the extent of resection (EOR), because the presence of residual glioma at either the initial (p = 0.007) or second (p = 0.001) surgery was associated with significantly shorter OS (28). The EOR did not differ between initial surgery and reoperation, with a mean EOR from 72% to 94% and a mean tumor residual volume from 3.1 to 8.8 ml (27, 29, 34, 36–38). Therefore, the benefit of repeat operations on survival is likely related to the cytoreduction effect, capable to delay DLGG transformation, as already demonstrated after the first surgery (2). Indeed, in the study of Shofty et al. (35), the median time to transformation was significantly prolonged after reoperation (14.4 years) compared with a control group without the second surgery (3.5 years, p = 0.0002) (38). However, the rate of MT histologically confirmed at reoperation is high, i.e., in a range between 19% and 74% in the literature (24–26, 31, 35, 36, 39), with a lesser EOR (37) and a decreased OS when reoperation is performed after tumor transformation (28).

Interestingly, molecular subtypes were not associated with significant differences in malignant progression-free survival or in OS in several series (28, 37), whereas two other studies suggested that multiple surgeries delay tumor recurrence, MT, and prolong OS in DLGG, with a more significant impact in IDH-mutated gliomas (29, 35).

### Functional Considerations

From a functional perspective, repeated surgery does not increase the risk of complications in comparison to the primary operation, by considering traumatic (related to the surgical manipulation), cerebrospinal fluid–related (leaks, hydrocephalus), septic, hemorrhagic, ischemic, epileptic, and general (non-neurological) factors (33, 40). In series specifically dedicated to DLGG, beyond the fact that no mortality was reported, all experiences supported the safety of multiple resections, with a low rate of permanent neurological impairment between 0% and 8.5%, similar to the morbidity rate of the first surgery (25, 26, 28–30, 32, 34–37). These favorable outcomes can be achieved with a high level of reproducibility regardless the DLGG location, including in challenging brain areas such as in eloquent sensorimotor and language structures (30, 32, 36) and in the insula (29, 34). Remarkably, these results were obtained because of the use of intraoperative electrical mapping in awake patients in most cohorts (25, 29, 30, 32, 34, 36–38).

Moreover, two recent series investigated cognitive outcomes by performing an extensive neuropsychological before and after repeat awake surgery in patients with DLGG (36, 38). Capo et al. (36) found a global preservation of the level of performance in 40 patients, despite changes in phonological fluency. Ng et al. (38) examined 62 patients, of which eight (12.9%) experienced a cognitive deficit before reoperation: 3 months following reoperation, four additional patients (6.5%) had a cognitive worsening, whereas eight (12.9%) patients improved in comparison with the preoperative status—the others were stable. Interestingly, the cognitive scores were not correlated to the EOR, knowing that the total or subtotal resections were performed in 91.9% of patients (mean EOR of 90.3%) (38). These findings support that multiple surgeries with awake mapping can be achieved with a large glioma removal and with an early recovery of neuropsychological abilities (38).

Multiple surgeries may even participate in improving QoL in patients who experienced more intense and/or frequent seizures at tumor relapse, by controlling epilepsy following reoperation (25). Another parameter critical for QoL is the capability to resume socio-professional activities. The rate of return to work was evaluated between 84.2% (25) and 94.2% (38) after the second surgery and at 84.6% after the third surgery (37), showing that the number of resections does not enhance the risk not to be able to resume an active life.

Thus, such data that show that iterative surgeries prolong OS while preserving QoL plead in favor of considering reoperation in a more systematic manner in DLGG (38, 41).

## When to Reoperate: Spatiotemporal Parameters Guiding the Decision in Reprogressive DLGG

### Iterative Operations and Neuroplastic Potential

Despite infiltration of DLGG within the brain, reoperation(s) with optimization of the onco-functional balance can nonetheless be performed because of the mechanisms of neural reallocation elicited by the slow tumor progression over years (42, 43). An increase of both the gray matter volume and functional connectivity of the contralesional homologous areas was evidenced at diagnosis using non-invasive neuroimaging (44, 45). These insights into the individual pattern of structural and functional neuroplasticity are critical to tailor the therapeutic strategy, especially regarding surgical indication and planning (46). The principle is to achieve a connectome-guided surgery in awake patient, with intraoperative mapping enabling to adapt the resection according to the redistribution of the neural networks that occurred in the preoperative period (47). Postoperative cognitive rehabilitation is also able to generate further degrees of cerebral reorganization, allowing functional recovery after a transitory worsening that may occur immediately following resection (48). Furthermore, in case of DLGG reprogression, the brain will continue to reshape, as demonstrated by longitudinal functional neuroimaging investigations (49, 50).

Therefore, this additional neural redeployment opens the door to subsequent surgical resection(s), which can be based on the connectome modifications that had taken place since the initial operation (47). Indeed, when comparing results of the awake mapping performed during reoperation with those obtained during the first surgery, changes in the functional organization were detected (30, 32). Such a remapping has been made possible due to the slow kinetics of recurrent DLGG, as illustrated by the long delay between the initial and second surgery, i.e., with a mean interval between 3 to 6.75 years (24, 25, 29, 30, 32, 35–38). This neuroplastic potential explains why the EOR does not significantly differ between the first surgery and reoperation (27, 29, 34, 36, 37), or can even been greater with no additional neurological or neurocognitive deficit (38). It may also explain why the time needed to recover independence can be significantly shorter at reoperation than after the first surgery (32).

Remarkably, this reshaping process could be similar following a second surgery, namely, with the possibility for the brain to continue to adapt in case of new DLGG relapse: this may permit to achieve a third operation several years later (mean interval of 5.4 years) with a similar EOR while maintaining QoL due to additional mechanisms of functional reallocation that arose in the meantime, as evidenced by awake mapping (37).

### Limitations

This concept of multistage surgical approach may, nonetheless, have limitations related to the spatiotemporal pattern of DLGG relapse: The therapeutic management should be tailored accordingly (51, 52).

Regarding spatial considerations, recent atlases of neuroplasticity have evidenced that the main limitation of neural redistribution is represented by the subcortical connectivity (53–55). Therefore, if the recurrent DLGG after initial surgery exhibits a more migratory pattern, with a prominent diffusion along the white matter tracts, then the EOR has a high risk to be less at reoperation (32). Such a connectomal constraint plays a major role in the decision to reoperate (or not), because the plastic potential is low at the level of the axonal fibers (56)—conversely to a high plastic potential at the level of the cortex, thus pleading in favor of iterative surgeries for tumor relapse with a more bulky pattern and that mainly involves the cortical areas (32, 57).

Concerning the temporal considerations, because neuroplasticity is dependent on the time course of the lesion, i.e., with a higher potential of compensation in reaction to slow-growing tumor (43), in case of acceleration of the glioma kinetics due to MT, the EOR can be less if one would like to preserve QoL (37). Similarly, rapid reprogression following initial resection (less than 1 to 2 years) was correlated with a shorter time to MT and a decreased OS (35, 51). Thus, these findings suggest a window for treatment opportunity, i.e., to propose reoperation earlier in case of DLGG reprogression, before the tumor transformed in a higher grade of malignancy (25, 37). To this end, the occurrence of a hypermetabolic focus on a longitudinal multimodal imaging study (such as repeat MRI perfusion and spectroscopy and/or F-DOPA Positron Emission Tomography PET) (58) may represent an additional argument in favor of redo surgery, even in the absence of gadolinium enhancement. A “prophylactic” reoperation can especially be discussed in specific circumstances, for example, if a woman had a desire for motherhood after a partial surgical resection of DLGG—because of a higher risk of MT and death following pregnancy in case of incomplete tumor removal (22).

## How to Reoperate: Adapting the Therapeutic Strategy to the Individual Glioma-Brain Interplay and to the Patient’s Needs

**Figure 1:**
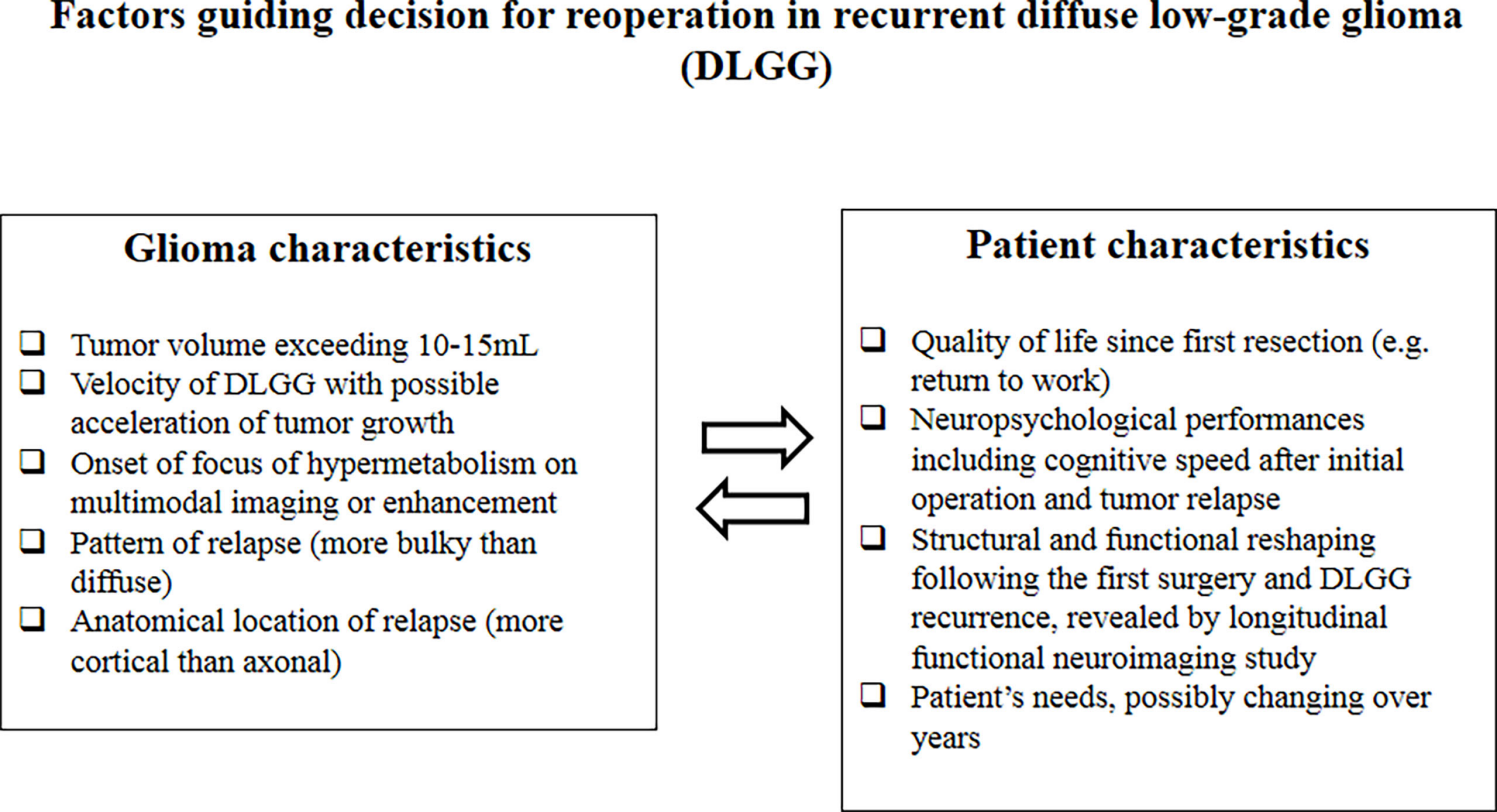
Factors guiding decision for reoperation in recurrent diffuse low-grade glioma (DLGG).

In addition to the considerable structural-functional variability across patients (57), the connectome is changing over time for each patient, based on constant glioma-brain dialogue (59, 60). This is particularly true at the level of the peritumoral zone, i.e., at the interface between the glioma core and the healthy brain, where intercommunication across tumoral cells and neural networks is maximal and where glioma relapse occurs most frequently (61). Cerebral circuits reconfiguration in reaction to the behavior of DLGG (pattern of proliferation versus migration, velocity diametric expansion) itself with constant modifications (spontaneously or consecutively to treatments) is possible, owing to the dynamics within and between networks in the framework of a meta-networking (network of networks) organization of brain processing (62). Surgical strategy should be adapted to this perpetual connectomal instability by detecting and preserving critical cortical hubs, often reallocated before (re)operation(s), as well as white matter tracts, because of direct electrostimulation (63). Because of frequent functional reshaping between the first and second surgery, or even between the second and third surgery, it is highly recommended to awake the patient to benefit not only from an accurate electrical mapping but also from an extensive cognitive and emotional monitoring performed in real time throughout the resection (12). With the aim of enhancing the sensitivity of such an actual neuropsychological evaluation into the operating theater, which should also take into consideration the spatial relationships between DLGG and surrounding eloquent pathways according to the tumor location (64, 65), the use of a multitasking protocol has been suggested, because it necessitates to recruit more neural circuits by increasing the cognitive demand—as a mirror of the meta-network (66). This protocol consists of a constant multitasking combining several tests performed simultaneously, during the transient presentation of a problem to solve on a computer screen while enabling to stimulate a specific cerebral structure in this time window, for example, movement combined with semantic association task while naming the pictures (66). Tasks should be selected on the basis of the expectations of the patient (e.g., monitoring of sensorimotor, language, visuospatial, executive, or behavioral functions), according to his/her familial, social, and professional activities as well as his/her environment (12). Nonetheless, in case of iterative surgeries, the patient’s wishes can change over years due to an evolution of the lifestyle. For example, at the time of the initial surgery, a 60-year-old patient may want to preserve a high level of executive functions because he/she is working full time, whereas at the time of reoperation several years later, the patient can be retired, with less requirement regarding higher-order cognition. In other words, the onco-functional balance should be reweighted at each moment with the ultimate aim to tailor a multistep medico-surgical management taking account of complex interactions between changes in patients’ needs, functional connectome, and DLGG course (67) (**Figure 1**).

Repeat operations must be integrated in a more global multimodal therapeutic attitude, which also comprises chemotherapy and radiotherapy. In case of rapid postoperative DLGG reprogression, early reoperation should be considered if functionally feasible, with adjuvant treatment to be performed immediately after the last surgery. Conversely, if the regrowth is slow after the initial resection, then postponing adjuvant therapies can be proposed, especially concerning radiotherapy (67). Indeed, irradiation of the white matter tracts has a risk to generate delayed cognitive deteriorations (68, 69). Moreover, radiotherapy-induced alterations in the brain microenvironment also contribute to recurrent glioma aggressiveness (70). Therefore, in the event of a slow DLGG reprogression following the first surgery, particularly in case of large resection, a simple surveillance may be considered on the basis of regular MRI control with volumetric measurement and calculation of growth rate (23, 71)—including in DLGG with foci of MT in the middle of the tumor (72). However, although reoperation should be preferred at recurrence, when the DLGG relapse exhibits a more migratory pattern along the white matter tracts, because EOR has a high risk to be less (32), chemotherapy may be discussed (67). Beyond the fact that global QoL is usually preserved (73), chemotherapy may induce a tumor shrinkage with a lesser degree of infiltration of the subcortical fibers, then reopening the window to a reoperation with improvement of the EOR (74, 75). The same principle can be applied after a second or even a third surgery, allowing to reach an OS close to 18 years (37).

Finally, although histo-molecular aspects must also be incorporated in this complex equation for managing DLGG recurrence (76), they represent only a part of the story (77), in addition to the functional and radiological parameters (67)—especially taking account of modifications of the genetic profile which may arise at DLGG relapse. Indeed, although IDH1mutation represents the earliest genetic alteration in DLGG, longitudinal analyses at recurrence found a high mutational potential, especially with possible clonal expansion and epigenetic reprogramming after deletion or amplification of mutant IDH1 (78). These preliminary data show that multiple operation(s) may represent a unique opportunity to better investigate the spatiotemporal heterogeneity of DLGG.

## Conclusions and Perspectives

As previously reported in high-grade glioma (79, 80), repeat surgery represents a safe and efficient therapeutic approach to enhance OS while preserving QoL in patients with DLGG. Favorable outcomes described in the recent literature support the proposal of early “preventive” reoperation in recurrent DLGG, before the onset of symptoms (e.g., seizures and cognitive or behavioral changes) and before MT.

These findings, however, may be limited by an intrinsic bias, namely, the patient selection, because by definition, reoperation was considered only when functionally feasible. Therefore, the next step would be to increase the number of surgical indications. To this end, a better prediction of the neuroplasticity reserve at every moment for each patient, based on an improved understanding of the perpetual interactions between the neural circuitry and the glioma course, would result in an optimization of the multistage and multimodal personalized management due to the anticipation of the next treatment(s) before DLGG transformation and/or onset of functional worsening. Integrating the concept of meta-plasticity (plasticity of the synaptic plasticity), i.e., a higher-order plastic phenomenon that regulates the learning rule as a function of the dynamical context (81), may help to reorient the spatiotemporal pattern of network reconfiguration (67). The principle would be to redirect the mechanisms of brain reshaping to generate a shift from a prior pattern with prominent perilesional recruitment (thus preventing to increase the EOR at the periphery of the previous surgical cavity) to a pattern predominantly recruiting remote circuits—ideally relying on contralateral homotopic structures (82). In this spirit, the concept of neuromodulation-induced cortical reallocation by using non-invasive transcranial stimulation tools starts to be developed, both for postoperative neurorehabilitation and for prehabilitation before (re)operation (83–85).

## Author Contributions

The author confirms being the sole contributor of this work and has approved it for publication.

## Conflict of Interest

The author declares that the research was conducted in the absence of any commercial or financial relationships that could be construed as a potential conflict of interest.

## Publisher’s Note

All claims expressed in this article are solely those of the authors and do not necessarily represent those of their affiliated organizations, or those of the publisher, the editors and the reviewers. Any product that may be evaluated in this article, or claim that may be made by its manufacturer, is not guaranteed or endorsed by the publisher.
